# Historical Gaps in the Integration of Patient-Centric Self-Management Components in HFrEF Interventions: An Umbrella Narrative Review

**DOI:** 10.3390/jcm14082832

**Published:** 2025-04-19

**Authors:** Pupalan Iyngkaran, Fareda Fazli, Hayden Nguyen, Taksh Patel, Fahad Hanna

**Affiliations:** 1 Melbourne Clninical School, University of Notre Dame, Melbourne, VIC 3030, Australia; pupalan.iyngkaran@student.torrens.edu.au; 2 Program of Public Health, Department of Health and Education, Torrens University Australia, Melbourne, VIC 3000, Australia; fareda.fazli@student.torrens.edu.au; 3 HeartWest, Werribee, VIC 3029, Australia; haydennguyen06@gmail.com (H.N.); taksh.patel@student.unsw.edu.au (T.P.)

**Keywords:** chronic disease self-management, clinical trials, congestive heart failure, self-management, narrative review, umbrella review, synthesis of evidence

## Abstract

**Background:** Chronic disease self-management (CDSM) interventions have shown promise in improving patient outcomes in heart failure (HF), particularly for those with reduced ejection fraction (HFrEF). Patient-centric self-management programs often incorporate key components such as education, self-monitoring, and goal setting. However, the extent to which these components are consistently reported and integrated into studies remains unclear. This umbrella narrative review aims to analyze systematic reviews to assess the consistency of reporting on patient-centric self-management components implemented in trials and studies. **Methods:** This umbrella narrative review synthesized findings from systematic reviews and meta-analyses published between 2000 and 2023 for CDSM tools in HF. Eligible studies were assessed for the presence and consistency of reporting on education, self-monitoring, and goal setting in self-management interventions for HFrEF. Data extraction focused on the frequency of reporting these components and the gaps in reporting long-term patient outcomes. **Results:** Among the included systematic reviews, education was the most consistently reported component (100%), while self-monitoring and goal setting were each reported in around 50% of studies. Reporting of long-term outcomes, such as mortality and quality of life, was highly variable and often absent. These inconsistencies highlight significant gaps in the evidence base for CDSM interventions. **Conclusions:** This review identifies gaps in the consistent reporting of key CDSM components in systematic reviews of HFrEF interventions. The inconsistent inclusion of all three components together and limited reporting of long-term outcomes may hinder the development of a robust evidence base for the adoption of these tools in HF guidelines. Future studies should prioritize comprehensive reporting to strengthen the foundation for patient-centric self-management strategies in HF care. **PROSPERO** registration number CRD42023431539.

## 1. Introduction

Heart failure with reduced ejection fraction (HFrEF) is a chronic, progressive condition that requires effective long-term management to improve patient outcomes and reduce hospitalizations. Comprehensive care for HFrEF involves both pharmacological treatments and patient-centered self-management strategies. These strategies aim to empower patients, encouraging active participation in their own care through patient education, self-monitoring, and goal setting. Such interventions are thought to improve self-management behaviors and help patients better manage symptoms, medications, and lifestyle changes [1].

While evidence supports the potential of self-management interventions for heart failure (HF) management, there is significant heterogeneity in the way these interventions are designed and implemented. Multicomponent interventions, which combine education, self-monitoring, and goal setting, are considered optimal for managing chronic diseases, as they address various aspects of self-management, improve patient engagement, and lead to better outcomes, including adherence, self-management behaviors, and quality of life [2,3,4,5,6]. However, despite the growing recognition of their importance, the extent to which all relevant components are integrated into self-management interventions for HFrEF remains unclear in the existing literature. Furthermore, psychosocial support and personalized care, both of which have been shown to be important for enhancing patient engagement and adherence, are often not included, or inadequately integrated into HF self-management interventions.

This umbrella narrative review aims to identify and reflect on the combinations of components of patient-centric self-management interventions that have previously been implemented for HFrEF. We focus on their inclusion in combination with stated outcome measures. Specifically, the absence or incomplete inclusion of critical components such as patient education, self-monitoring, goal setting, and psychosocial patient and carer support may have contributed to the historical lack of comprehensive evidence for the adoption of chronic disease self-management tools in clinical guidelines. Our goal is to identify these gaps in the existing literature, where interventions may have fallen short, and to explain how addressing these gaps could enhance the future development of clinical guidelines for managing HFrEF.

## 2. Methods

### 2.1. Study Design

This paper is designed with an umbrella narrative format and synthesizes findings from systematic reviews and meta-analyses to explore the inclusion of key components in patient-centric self-management interventions for patients with HFrEF. The methodology and systematic review on efficacy have previously been published [7,8]. The current review does not aim to assess the effectiveness of interventions, but rather, it advances the topic in a narrative fashion and aims to expose gaps in the integration of essential components such as patient education, self-monitoring, goal setting, and psychosocial support, which are critical to comprehensive chronic disease management. By analyzing the components included in these interventions, we seek to understand how the absence or incomplete inclusion of these elements may have impacted the current evidence base and the historical adoption of self-management tools in clinical guidelines and generate hypotheses [9,10,11,12,13,14,15,16].

### 2.2. Eligibility Criteria

We included systematic reviews and meta-analyses published between 2000 and 2023 that assessed the effects of self-management interventions for HFrEF patients. The interventions must have included at least two of the following components: patient education, self-monitoring, goal setting, and psychosocial support. Studies that focused on interventions without these core elements, or those not specifically targeting HFrEF, were excluded. Reviews that did not provide sufficient details on the components of the interventions or the reporting of outcomes were also excluded [7].

### 2.3. Data Sources and Search Strategy

We conducted a comprehensive search across multiple databases, including PubMed, Cochrane Database of Systematic Reviews, Web of Science, Embase, and CINAHL, using the following keywords: “heart failure with reduced ejection fraction”, “self-management interventions”, “patient education”, “self-monitoring”, “goal setting”, and “psychosocial support”. The search was limited to English-language articles published from 2000 to 2023 [7].

### 2.4. Data Extraction

Two independent reviewers screened the systematic reviews and meta-analyses for eligibility (TP and PI) based on predefined inclusion and exclusion criteria. Relevant data were extracted, including (i) study characteristics: author, year of publication, sample size, study design, and participant demographics; (ii) intervention components: details of the self-management interventions included in the studies, focusing on whether patient education, self-monitoring, goal setting, and psychosocial support were part of the intervention; (iii) outcome measures: the outcomes measured in each study, particularly focusing on self-management behaviors, hospital readmissions, quality of life, and psychosocial outcomes; (iv) study quality: the quality of the systematic reviews and meta-analyses was assessed using the AMSTAR-2 tool [7,17], which evaluates methodological rigor in systematic reviews.

### 2.5. Synthesis of Results

Data were synthesized qualitatively to assess the presence or absence of the key components in the included self-management interventions. We focused on identifying the most commonly included components and exploring how their absence or incomplete inclusion might have influenced the reported outcomes. Given the heterogeneity in study designs and outcomes, a narrative synthesis was performed rather than a statistical meta-analysis. Studies were divided into pre 2014 (*Historical gaps*) and post 2014 (Umbrella Narrative review “*UmbNR*”) based on a decade of new articles, the demotion of CDSM grading as an HF performance measure [4], and the subsequent shaping of the 2022 HF guidelines [1].

### 2.6. Definitions of Key Terms

To ensure clarity, the following key terms related to patient-centric self-management interventions are defined in Table 1 (Appendix A, Table A1 glossary).

## 3. Results

### 3.1. Study Characteristics

A total of 60 systematic reviews and meta-analyses were included, covering a total of 276,381 participants. Interventions ranged from single component to multicomponent self-management strategies. The studies assessed a range of self-management interventions focused on patient education, self-monitoring, and goal setting, with varying intervention formats, including telehealth, patient education programs, self-monitoring tools, and carer education and support. The included systematic reviews and meta-analyses were diverse in their design, populations, and intervention types. Appendix A, Table A1 provides an overview of the key characteristics (from *Historical Gaps* and *UmrNR*) of these studies, including sample sizes, study durations, and reported outcomes. The predominant management difference in *historical gap* and *UmbNR* studies involved the use of newly approved therapeutics and novel technologies. The main study related aims for the *UmbNR* are highlighted below.

### 3.2. Umbrella Narrative Review

i. *Intervention Components*

The most used intervention component was patient education, which appeared in 100% of the included reviews. Self-monitoring and goal setting were also frequently used, appearing in 50% of the studies. Interventions describing education, support, and the patients’ carers were around 15% (Table 2).

ii. *Outcome Measures*

The studies assessed a variety of outcomes, with the most measured outcomes being quality of life and self-management behaviors, followed by readmissions, hospitalizations, and finally, in only 47.5%, mortality. The frequency of these outcomes is described in Table 2 and Table 3.

iii. *Frequency of Intervention Components*

An assessment of intervention components revealed variability in the inclusion of critical self-management elements. As shown in Table 2 and Table 3, patient education was the most frequently included component (100%), while self-management, including components of self-monitoring, goal setting, psychosocial support, and carer involvement were included in only 50% and 15% of studies, respectively. This variability highlights gaps in the integration of essential patient-centric elements. To provide additional context Table 3 and Table A1 (from Historical Gaps) expands on the intervention types, categorizing them into single- and multicomponent approaches. It shows that multicomponent interventions, while more comprehensive, were inconsistently implemented across *UmbNR* studies.

iv. *Outcome Reporting*

The outcome measures reported across the studies varied significantly. Table 2 presents a breakdown of the outcomes, such as self-management behaviors, hospital readmissions, and quality of life. However, long-term outcomes such as mortality were reported in less than 47% of studies, as noted in Table 1 (from Historical Gaps), which provides additional insights into the endpoints and their frequency of reporting, where mortality was reported at 65%.

### 3.3. Study Quality and Gaps

Study quality, evaluated using the AMSTAR-2 tool, is summarized in Table 4. Approximately 66% of studies were rated as moderate-to-high quality. However, gaps in reporting intervention components, especially psychosocial and carer support, and the lack of standardized outcome measures were common across studies. These gaps may partially explain the historical challenges in integrating CDSM tools into clinical guidelines.

## 4. Discussion

This umbrella narrative review consolidates findings from several systematic reviews and meta-analyses examining the effectiveness of patient-centric self-management interventions for patients with HFrEF [8]. The findings underscore the missed approaches to multicomponent interventions that incorporate patient education, self-monitoring, and goal setting in improving self-management behaviors and reducing hospital readmissions. These interventions, if paired with optimized measures, telehealth and psychosocial support, may enhance patient engagement and support sustainable self-management [1,3,4,6,78,79,80,81,82].

If the inclusion of these essential components is critical for HF outcomes, then our analysis reveals substantial gaps in the existing evidence base [3,18,19,20,21,22,23,24,25,26,27,28,29,30,31,32,33,34,35,36,37,38,39,40,41,42,43,44,45,46,47,48,49,50,51,52,53,54,55,56,57,58,59,60,61,62,63,64,65,66,67,68,69,70,71,72,73,74,75,76,77,80,83]. A key gap is the incomplete integration of all relevant patient-centric components. Despite the prevalence of patient education in the included studies, psychosocial support, a crucial factor in fostering patient adherence and engagement, was notably absent in many interventions. The heterogeneity of the included studies, with varying intervention designs and outcome measures, further suggests that it may be incorrect that chronic disease management tools have limited capacity if all their subcomponents are not adhered to; hence well designed trials are needed to evaluate the totality of their effectiveness.

The multicomponent interventions that we think would be most effective, including education, self-monitoring, and goal setting, have been shown historically to be most frequently associated with improvements in self-care behaviors, such as medication adherence and symptom monitoring. Indeed, previous studies suggest that interventions targeting multiple aspects of self-management tend to be more effective than single-component programs [5,7]. However, the absence of consistent reporting on long-term outcomes, such as mortality and sustained improvements in quality of life, hinders the ability to assess evidence for clinical uptake into practice.

### 4.1. Psychosocial Factors and Patient-Centered Care

One of the critical gaps identified in this review is the lack of integration of psychosocial factors in the intervention design. As noted by Lee and Villero (Table 3), mental health issues, including anxiety and depression, significantly influence a patient’s ability to engage in self-management behaviors. Addressing these factors through psychosocial support could improve patient adherence and quality of life, especially in populations at higher risk of poor health outcomes. Incorporating personalized care tailored to the psychosocial and emotional needs of patients with chronic diseases, including HF, is essential for optimizing self-management strategies. There remains an underappreciation for the burden of HF and chronic diseases on psychological health. The INTERHEART study across 52 countries and in excess of 27,000 participants attribute risk of at least 25% of mental health in preventing primary myocardial infarction in older and even higher (>43%) in younger patients [84]. The impact of chronic diseases on mental health is underappreciated. For instance, the INTERHEART study (a study of risk factors for first myocardial infarction in 52 countries and over 27,000 subjects) reported an attributable risk of psychosocial risk factors in the primary prevention of myocardial infarction of 25.2% in older patients (men > 55 years of age, women >65 years of age) and of 43.5% in younger patients. When established intervention can influence this outcome, it is no surprise some advocate for paradigm changes in our approach [85]. Finally, the influences and determinants of psychosocial health are broadly focusing on the self-management capabilities generically will cover this need. Adding the concept of dyads broadens the patients who can receive this type of care and negates the influence of some modifiable determinants [86,87].

### 4.2. Quality and Discernible Evidence

The study further shows that consensus is difficult as the large body of published evidence has tremendous heterogeneity. The authors identify four areas of relevance to shape future models. Firstly, chronic disease strategies have sentinel links to Wagner’s models [1,2]. The study noted that historical high-quality trials [31,32,33,79,80,81,82,83,84] have well described DMPs, e.g., multidisciplinary, case management, or clinical models (Table 4). Program leads were most often nurses, doctors, and various allied health personnel, looking after inpatients, transitioned patients, or predominately ambulatory outpatients. The evidence of improvements is a mixture of qualitative and quantitative. Secondly, there is difficulty in sieving through evidence and weighing contributions of new technologies or models from newer studies. The high cost of new treatments entails a weighting must be used to judge value. Thirdly, newer studies appear to explore more sophisticated delivery methods and technologies. The studies were complex in their mechanisms and tended to receive low grades. Finally, we raise the point of the value of cost-effectiveness in health services research.

## 5. Limitations

A limitation of this umbrella narrative review is the reliance on published systematic reviews and meta-analyses, which may introduce publication bias. This design excludes unpublished studies or data, potentially overlooking relevant findings that could affect the overall synthesis of evidence. Despite these limitations, the review’s aim was to identify patterns in the existing evidence and highlight areas where future research could address these gaps. Technological advances in health applications, e.g., mobile or AI-based tools, can contribute to self-management improvements. These factors are difficult to quantify and factor in when comparing the historical and current studies. In addition, there are pleiotropic effects of HF drug therapies which can also influence this comparison, and could be factored [88].

## 6. Conclusions

**Figure 1:**
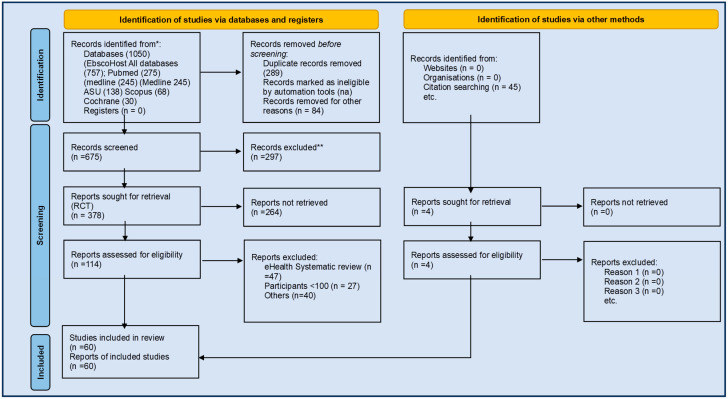
PRISMA 2020 flow diagram for new systematic reviews, which included searches of databases, registers, and other sources. Modified from: Page, M.J., et al., 2021 [9]. For more information, visit: http://www.prisma-statement.org/ (accessed on 1 June 2024).

This umbrella narrative review underscores the importance of patient-centric self-management interventions for improving outcomes in HFrEF patients, but it also highlights significant gaps that limit the applicability and endorsement of these interventions in clinical guidelines. Key gaps include inconsistent adding of inclusive self-monitoring and goal setting as well as inconsistent endpoint reporting and the absence of long-term data on outcomes like mortality and health-related quality of life. This variability likely influences inconsistent outcome measures across studies. Our review emphasizes that individual multicomponent patient education, self-monitoring, goal setting, and psychosocial support are the most effective approach for improving self-care behaviors when all are included. However, without all individual multicomponents, further research that accounts for semi-component interventions will remain limited in their ability to deliver consistent and meaningful improvements in HFrEF management. To bridge these gaps, future research must prioritize standardized patient centric self-care methodological approaches and practices that match consistent outcome measures, with long-term follow-up. This would help ensure that the benefits of self-management interventions can be more effectively realized, tailored to the diverse needs of patients, and ultimately endorsed by clinical guidelines (Figure 1).

## Figures and Tables

**Table 1:** Definition of key terms.

Term	Definition
Patient Education	Interventions aimed at increasing patient knowledge about their condition, including disease process, symptoms, and management strategies.
Self-Monitoring	The process of patients tracking their health status, such as weight, blood pressure, or symptoms, using tools like mobile health apps or written logs.
Goal Setting	Collaborative process where patients and providers set achievable, personalized goals related to managing their condition, such as medication adherence or lifestyle changes.
Self-Care Behaviors	Actions taken by patients to manage their condition, including medication adherence, symptom monitoring, physical activity, and dietary changes.
Telehealth	The use of digital communication platforms (e.g., phone calls, video conferencing) to provide healthcare services remotely, often used to support ongoing monitoring and education.

**Table 2:** Reported outcomes and frequency of the core components of self-management interventions from UmbNR compared to historical gaps.

Era	* Outcome Measure				@Intervention Component		
	Mortality	Hospitalizations	Self-Care Behaviors	Quality of Life	Patient Education	Self-Monitoring	Carer Goal-Setting
UmbNR Post 2014 (*n* = 40)	19/21	26/14	35/5	32/8	40/40	20/40	6/40
Historical Gaps Pre 2014 (*n* = 20)	13/7	13/7	17/3	15/5	20/20	12/20	5/20
Frequency (%)	47.5/65	65/65	87.5/85	80/75	100/100	50/60	15/25

* Presented as the number of studies that reported the outcome as either positive, equivocal, or negative versus not reported. @ Presented as the number of studies that reported each intervention component in the methodology and results over the total number of included studies.

**Table 3:** Frequency of core components in chronic disease self-management models as reported from UmbNR and historical gaps studies.

Author (Year); Country	Population Identification Process (>60 HF <40%)	Evidence-Based Practice Guidelines (>50% Describe Guidelines)	Collaborative Practice Models >50%	Patient Self-Management Education (Defined in >60% to <40%)	Process and Outcome Management (60> to >40% Protocols)	Reporting and Feedback Loop (Described in Study)	Strength of Evidence	Summary of Study Intervention	Notes
Zhao et al., 2024 China [18]	-	~	+	++	+	+	L	Case Management 4 MDT care models	HF population, care model not clear, FU variable
Chen et al., 2023 Taiwan [19]	-	~	++	-	+	+	L	Case Management Collaborative Health Management	HF Class provided but not HFrEF or HFpEF details
Li 2023 China [20]	++	~	+	-	++	+	L	Case Management Transitional Care	Minimal description of CDSM
Yang 2023 Mly [21]	+	~	+	-	+	+	L	Case Management Multicomponent Integrated Care	Limited description of models and processes
Hakams 2022 Holland [22]	++	~	++	++	++	++	H	Case Management Care Pathways	Umbrella SR. Includes all treatments 67/146 RCT on care pathway
Hsu 2022 Taiwan [23]	+	~	+	+	-	+	L	Case Management Patient Navigators	Many limitations in the pooling of information
Toback 2017 Canada [24]	-	~	+	+	-	-	L	Case Management Multiple SM support	Good SM information, but borderline criteria for SR and inclusion.
Taylor 2005 UK [25]	++	~	++	++	++	++	H	Case Management DMP (MDT, CMM, CM)	Multiple disease management models. Indeterminate
Roccaforte 2005 Canada [26]	++	~	++	++	++	++	H	Case Management DMP	DMP improve MACE.
Gonseth 2004 Spain [27]	++	~	++	++	++	++	H	Case Management DMP—elderly	DMP improve MACE esp. elderly.
Huang 2023 China [28]	-	~	+	+	-	-	M	Nurse-Led SM	Poorly descriptive meta-analysis
Nwosu 2023 UK [29]	-	~	+	-	-	-	L	Nurse-Led Patient Education	Poorly descriptive. Outcomes data focus
Checa 2022 Spain [30]	++	~	++	++	++	++	H	Nurse-Led Case management primary care	Primary care study with cost-effectiveness
Huang 2022 China [31]	-	~	++	++	++	++	M	Nurse-Led SM	Good study. HF diagnosis is not clear.
Ceu 2022 Portugal [32]	-	~	-	-	-	-	VL	Nurse-Led Variable nursing interventions	Brief general discussion on studies.
Imanuel Tonapa 2022 Taiwan [33]	++	~	++	+	++	++	M	Nurse-Led Telecoaching	Self-management programs not well described.
Son 2020 Sth Korea [34]	++	~	++	++	++	++	M	Nurse-Led SM education	Good study. Minor gaps for high grade.
Walsh 2017 Ireland [35]	-	~	-	+	-	-	L	Nurse-Led Clinic based SM education	Limited information in many areas.
Alnomasy 2023 USA [36]	-	~	++	++	++	+	M	Non-Pharmacological Ambulatory—home visits, phone calls, digital platforms, technologies.	HF not well characterized.
Mhanna 2023 USA [37]	++	~	++	++	++	++	H	Non-Pharmacological CBT	Depression focus on CDSM.
Olano-Lizarraga 2023 Spain [38]	++	~	++	++	++	++	M	Non-Pharmacological Interventions targeting the social dimension	Good study. Minor gaps for high grade
Nso 2023 USA [39]	-	~	+	+	-	-	L	Non-Pharmacological CBT	Poorly descriptive. Outcomes data focus
Balata 2023 Germany [40]	++	~	++	++	++	++	M	Non-Pharmacological CBT	Good study. Depression focus.
Koikai 2023 UK [41]	+	~	++	++	++	++	M	Non-Pharmacological SM education strategies	Some studies did not report HF grade adequately.
Feng 2023 China [42]	++	++	++	++	++	++	M	Non-Pharmacological SM intervention strategies	Only study to describe guideline utilized.
Nahlen Bose 2023 Sweden [43]	-	~	++	+	++	++	M	Non-Pharmacological Psychosocial Interventions	Good study some gaps in HF and SM details.
Lee 2022 USA [44]	++	~	++	++	++	++	H	Non-Pharmacological SM intervention	Excellent focused, some data extrapolated from citation.
Villero-Jimenez 2022 Spain [45]	+	~	++	++	++	++	M	Non-Pharmacological Dyadic SM interventions	Spanish translated. Gaps in HF details
Ghizzardi 2022 Italy [46]	++	~	++	++	++	++	H	Non-Pharmacological Motivational interviewing on SM	Focus on delivery programs.
Suksatan 2022 Thailand [47]	+	~	++	++	++	++	M	Non-Pharmacological Transitional Care Intervention elderly	Excellent description of care programs. HF details lacking.
Meng 2021 China [48]	-	~	++	++	++	++	M	Non-Pharmacological SM intervention	Covered all domains, but studies specifics lacking.
Tinoco 2021 Brazil [49]	-	~	++	++	-	-	L	Non-Pharmacological Health education and SM	Scoping nature, limited details.
Aghajanloo 2021 Iran [50]	++	~	++	++	++	++	M	Non-Pharmacological SM behaviors with SCHFI	Good description on CDSM.
Cañon-Montañez 2021 Colombia [51]	-	~	++	++	++	++	M	Non-Pharmacological Educational Intervention	Superficially covers all domains.
Anderson 2021 UK [52]	-	~	++	++	++	++	L	Non-Pharmacological Advanced-level nurses specialist nurse-led vs. physician-led	It covers broad areas superficially.
Zhao 2021 China [53]	-	~	++	++	++	++	M	Non-Pharmacological SM interventions	Excellent study, few HF details.
Poudel 2020 USA [54]	-	~	++	++	++	++	M	Non-Pharmacological Motivational interviewing	Covers relevant domains.
Świątoniowska-Lonc 2020 Poland [55]	++	~	++	+	+	++	VL	Non-Pharmacological Health Education	It covers many domain in very superficial detail.
Peng 2019 China [56]	++	~	+	+	+	++	L	Non-Pharmacological CBT	Focused area, outcomes strong point.
Parajuli 2019 Australia [57]	++	~	++	++	++	++	H	Non-Pharmacological Pharmacist Involved MDT	High quality study, few flaws.
Shanbhag 2018 Canada [58]	-	~	++	+	++	++	L	Non-Pharmacological Interventions improving physician adherence to guideline	Variable study design. Poor HF description.
Sterling 2018 USA [59]	-	~	++	+	++	++	L	Non-Pharmacological Home Care Workers	Missing important data.
Jiang 2018 Taiwan [60]	-	~	+	+	+	++	M	Non-Pharmacological SM Psychological Interventions	Good study, focus on meta-analyses. Domain description reduced.
Jonkman 2016 Holland [61]	++	~	++	++	++	++	H	Non-Pharmacological SM, program details	Gold-standard.
Ruppar 2016 USA [62]	++	~	++	++	++	++	H	Non-Pharmacological Medication adherence.	Detailed, informing study.
Jonkman 2016 Holland [63]	++	~	++	++	++	++	H	Non-Pharmacological SM interventions	Gold-standard
Srisuk 2016 Thailand [64]	++	~	++	++	++	++	L	Non-Pharmacological Family based education	Detailed. Quality influenced by grading criteria
Ha Dinh 2016 Vietnam [65]	++	+	++	++	++	++	L	Non-Pharmacological Teach-back method, SM	Detailed study. Technical gaps.
Inglis 2015 Australia [66]	++	++	++	++	++	++	H	Non-Pharmacological Structured Telephone support, telemonitoring	Gold standard.
Ruppar 2015 USA [67]	++	~	++	++	++	++	H	Non-Pharmacological Medication adherence	Covers an important topic.
Casimir 2014 USA [68]	++	+	++	++	++	++	M	Non-Pharmacological Patient centered SM	Comprehensive.
Wakefield 2013 USA [69]	++	~	++	++	++	++	H	Non-Pharmacological Care Management Program	Descriptive.
Barnason 2012 USA [70]	++	~	++	++	++	++	M	Non-Pharmacological SM Interventions	Few flaws, grading based on scoring system.
Boyde 2011 USA [71]	-	~	++	++	++	++	M	Non-Pharmacological Educational Interventions	Good SM study, details on HF lacking.
Dickson 2011 USA [72]	++	~	++	++	++	++	H	Non-Pharmacological SM Practices	Very limited high quality hypothesis generating study.
Yehle 2010 USA [73]	++	~	++	++	++	++	H	Non-Pharmacological Educational Interventions	Good SM study.
Ditewig 2010 Holland [74]	++	~	++	++	++	++	H	Non-Pharmacological SM Interventions	Good SM study.
Boren 2009 USA [75]	++	~	++	++	++	++	H	Non-Pharmacological SM education	Good SM study.
Jovicic 2006 Canada [76]	++	~	++	++	++	++	H	Non-Pharmacological SM intervention	Good SM study.
McAlister 2004 Canada [77]	++	~	++	++	++	++	H	Non-Pharmacological Multidisciplinary strategies	Good SM study.

Table 3 outlines in detail the reporting of six core chronic disease management domains utilized in models of care. High quality studies (H) were excellent is describing these domains, except HF guidelines. The domain referencing HF guidelines utilized were poorly or not described (-,~) in almost all the studies. The descriptions of the remaining four chronic disease model domains are in the table. *Reporting of chronic disease components*: (++) >60% of studies report; (+) 40–60% of studies report; (-) poorly reported <40% of studies; (~) no clear indication studies have reported this domain *Abbreviations:* CDSM—chronic disease self-management; CM—clinic model; CMM—case management model; DMP—disease management programs; FU—follow-up; HF—heart failure; HFrEF—heart failure with reduced ejection fraction, HFpEF—heart failure with preserved ejection fraction; H—High; L—low; Medium; VL—very low; MACE—major adverse cardiovascular outcomes; MDT—multidisciplinary team; SM—self-management; UK—United Kingdom; USA—United States of America.

**Table 4:** AMSTAR grading of articles and certainty of evidence (study quality and identified gaps).

Author (Year)	1	2	3	4	5	6	7	8	9	10	11	12	13	14	15	16	Total
Zhao et al., 2024 [18]	Y	PY	Y	PY	Y	Y	N	PY	Y	N	N-MA	N-MA	N	N	N-MA	Y	L
Chen et al., 2023 Tai [19]	Y	PY	Y	PY	Y	Y	N	Y	Y	N	Y RCT	Y	Y	Y	Y	Y	L
Li China 2023 [20]	Y	PY	Y	PY	Y	Y	N	Y	Y RCT	N	Y RCT	Y	Y	Y	N	Y	L
Yang Mly 2023 [21]	Y	PY	Y	PY	Y	Y	N	Y	Y RCT	N	Y RCT	Y	Y	Y	Y	Y	L
Hafkamp 2022 [22]	Y	PY	Y	Y	Y	Y	Y	Y	Y RCT	Y	Y RCT	Y	Y	Y	Y	Y	H
Hsu 2022 [23] L-Ch	Y	PY	Y	PY	Y	Y	Y	PY	PY	Y	Y	PY	PY	PY	N	Y	L
Toback 2017 [24]	Y	PY	Y	PY	Y	Y	N	Y	Y RCT	N	Y RCT	Y	Y	Y	Y	Y	L
Taylor 2005 [25]	Y	PY	Y	Y	Y	Y	PY	PY	Y RCT	Y	Y RCT	Y	Y	Y	Y	Y	H
Roccaforte 2005 [26]	Y	PY	Y	PY	Y	Y	PY	PY	Y RCT	Y	Y RCT	Y	Y	Y	Y	Y	H
Gonseth 2004 [27]	Y	PY	Y	Y	Y	Y	Y	Y	Y RCT	Y	Y RCT	Y	Y	Y	Y	Y	H
Huang 2023 [28]	Y	PY	Y	PY	Y	Y	Y	Y	Y	Y	Y	Y	Y	Y	Y	Y	M
Nwosu 2023 [29]	Y	PY	Y	PY	Y	Y	Y	PY	PY	Y	Y	PY	PY	PY	N	Y	L
Checa 2022 [30]	Y	Y	Y	Y	Y	Y	Y	Y	Y RCT	Y	Y RCT	Y	Y	Y	Y	Y	H
Huang 2022 [31]	Y	PY	Y	PY	Y	Y	Y	Y	Y RCT	N	Y RCT	Y	Y	Y	Y	Y	M
Ceu 2022 [32]	Y	PY	Y	PY	Y	Y	N	Y	N BOTH	N	N BOTH	N-MA	N	N	N-MA	N	VL
Imanuel Tonapa 2022 [33]	Y	PY	Y	PY	Y	Y	PY	Y	PY RCT	N	Y RCT	N	Y	Y	Y	Y	M
Son 2020 [34]	Y	PY	Y	PY	Y	Y	PY	Y	Y RCT	Y	Y RCT	Y	Y	Y	N	Y	M
Walsh 2017 [35]	Y	PY	Y	PY	Y	N	N	PY	N BOTH	N	N-MA	N-MA	N	N	N-MA	N	L
Alnomasy 2023 [36]	Y	Y	Y	PY	Y	Y	Y	Y	Y	Y	Y	Y	Y	Y	Y	Y	M
Mhanna 2023 [37]	Y	PY	Y	Y	Y	Y	PY	PY	Y RCT	Y	Y RCT	Y	Y	Y	Y	Y	H
Olano-Lizarraga 2023 [38]	Y	PY	Y	PY	Y	Y	N	Y	Y RCT	N	Y RCT	Y	Y	Y	N	Y	M
Nso 2023 [39]	Y	PY	Y	PY	Y	Y	N	Y	Y RCT	N	Y RCT	Y	Y	Y	Y	Y	L
Balata 2023 [40]	Y	Y	Y	PY	Y	Y	N	Y	Y RCT	N	Y RCT	Y	Y	Y	Y	Y	M
Koikai 2023 [41]	Y	PY	Y	PY	Y	Y	N	Y	Y BOTH	N	N	N-MA	N	N	N	Y	M
Feng 2023 [42]	Y	PY	Y	PY	Y	Y	Y	Y	Y	Y	Y	Y	Y	Y	Y	Y	M
Nahlen Bose 2023 [43]	Y	PY	Y	PY	Y	N	Y	Y	Y RCT	Y	Y RCT	Y	Y	Y	Y	Y	M
Lee/Reigel 2022 [44]	Y	PY	Y	PY	Y	Y	N	PY	Y RCT	N	Y RCT	Y	Y	Y	Y	Y	H
Villero-Jimenez 2022 [45]	Y	PY	Y	PY	Y	Y	Y	Y	Y	Y	Y	Y	Y	Y	Y	Y	M
Ghizzardi 2022 [46]	Y	PY	Y	Y	Y	Y	Y	Y	Y RCT	Y	Y RCT	Y	Y	Y	Y	Y	H
Suksatan 2022 [47]	Y	PY	Y	Y	Y	Y	Y	Y	Y BOTH	Y	Y RCT	Y	Y	Y	Y	Y	M
2021 Meng [48]	Y	PY	Y	PY	Y	Y	Y	PY	PY RCT	Y	Y RCT	N	Y	Y	Y	Y	M
Tinoco 2021 [49]	Y	PY	Y	PY	Y	Y	Y	PY	PY RCT	N	Y RCT	Y	Y	Y	Y	Y	L
Aghajanloo 2021 [50]	Y	PY	Y	PY	Y	Y	Y	PY	Y NRSI	N	Y BOTH	Y	Y	Y	Y	Y	M
Cañon-Montañez 2021 [51]	Y	Y	Y	Y	Y	Y	Y	Y	PY RCT	Y	Y RCT	Y	Y	Y	Y	Y	M
Anderson 2021 [52]	Y	PY		PY	Y	Y	PY	Y	Y BOTH	N	N BOTH	N MA	Y	Y	Y	Y	L
Zhao 2021 [53]	Y	PY	Y	PY	Y	Y	N	Y	PY RCT	N	Y RCT	N	Y	Y	Y	Y	M
Poudel 2020 [54]	Y	Y	Y	Y	Y	Y	Y	Y	PY RCT	Y	Y RCT	Y	Y	Y	Y	Y	M
Świątoniowska-Lonc 2020 [55]	Y	PY	Y	PY	Y	Y	PY	Y	PY RCT	Y	Y RCT	Y	Y	Y	N-MA	Y	VL
Peng 2019 [56]	Y	PY	Y	PY	Y	Y	PY	PY	Y RCT	Y	Y RCT	Y	Y	Y	N	Y	L
Parajuli 2019 [57]	Y	PY	Y	Y	Y	Y	Y	Y	Y RCT	Y	Y RCT	Y	Y	Y	Y	Y	H
Shanbhag 2018 [58]	Y	Y	Y	PY	Y	Y	Y	Y	Y BOTH	N	N-MA	N-MA	Y	Y	N-MA	Y	L
Sterling 2018 [59]	Y	Y	Y	PY	Y	Y	PY	Y	Y NRSI	Y	N-MA	N-MA	Y	Y	N	Y	L
Jiang 2018 [60]	Y	PY	Y	PY	Y	Y	PY	PY	Y RCT	Y	Y RCT	Y	Y	Y	Y	Y	M
Jonkman 2016 [61]	Y	PY	Y	PY	Y	Y	PY	Y	Y RCT	Y	Y RCT	Y	Y	Y	Y	Y	H
Ruppar 2016 [62]	Y	PY	Y	PY	Y	Y	N	Y	Y RCT	N	Y RCT	Y	Y	Y	Y	Y	H
Jonkman 2016 [63]	*Y*	*PY*	*Y*	*PY*	*Y*	*Y*	*PY*	*Y*	*Y RCT*	*Y*	*Y RCT*	*Y*	*Y*	*Y*	*Y*	*Y*	*H*
Srisuk 2016 [64]	Y	PY	Y	PY	Y	Y	PY	Y	Y RCT	Y	N-MA	N-MA	Y	Y	N-MA	Y	L
Ha Dinh 2016 [65]	Y	PY	Y	PY	Y	Y	Y	Y	Y BOTH	N	N MA	N MA	Y	Y	N-MA	Y	L
Inglis 2015 [66]	Y	Y	Y	PY	Y	Y	PY	PY	Y RCT		Y RCT	Y	Y	Y	Y	Y	H
Ruppar 2015 [67]	Y	Y	Y	PY	Y	Y	PY	Y	Y RCT	Y	Y RCT	Y	Y	Y	Y	Y	H
Casimir [68]	Y	PY	Y	Y	Y	Y	PY	PY	Y RCT	N	N-MA	N-MA	Y	N	N-MA	Y	M
Wakefield 2013 [69]	Y	PY	Y	Y	Y	Y	Y	Y	Y RCT	Y	Y RCT	Y	Y	Y	Y	Y	H
Barnason 2012 [70]	Y	Y	Y	Y	Y	Y	N	Y	Y RCT/PY NRSI	Y	Y BOTH	Y	Y	N	N	Y	M
Boyde 2011 [71]	Y	PY	Y	PY	Y	Y	Y	Y	Y	Y	Y	Y	Y	Y	Y	Y	M
Dickson 2011 [72]	Y	PY	Y	Y	Y	Y	Y	Y	Y RCT	Y	Y RCT	Y	Y	Y	Y	Y	H
Yehle 2010 [73]	Y	PY	Y	Y	Y	Y	Y	Y	Y RCT	Y	Y RCT	Y	Y	Y	Y	Y	H
Ditewig 2010 [74]	Y	PY	Y	PY	Y	Y	PY	Y	Y RCT	N	N-MA	N-MA	Y	Y	N-MA	Y	H
Boren 2009 [75]	Y	PY	Y	Y	Y	Y	Y	Y	Y RCT	Y	Y RCT	Y	Y	Y	Y	Y	H
Jovicic 2006 [76]	Y	PY	Y	Y	Y	Y	PY	Y	Y RCT	Y	Y RCT	Y	Y	Y	Y	Y	H
McAlister 2004 [77]	Y	PY	Y	PY	Y	Y	PY	Y	Y RCT	Y	Y RCT	Y	Y	Y	Y	Y	H

AMSTAR Questions: 1. Did the research questions and inclusion criteria for the review include the components of PICO? 2. Did the report of the review contain an explicit statement that the review methods were established prior to the conduct of the review and did the report justify any significant deviations from the protocol? 3. Did the review authors explain their selection of the study designs for inclusion in the review? 4. Did the review authors use a comprehensive literature search strategy? 5. Did the review authors perform study selection in duplicate? 6. Did the review authors perform data extraction in duplicate? 7. Did the review authors provide a list of excluded studies and justify the exclusions? 8. Did the review authors describe the included studies in adequate detail? 9. Did the review authors use a satisfactory technique for assessing the risk of bias (RoB) in individual studies that were included in the review? RCTs; NRSI 10. Did the review authors report on the sources of funding for the studies included in the review? 11. If meta-analysis was performed did the review authors use appropriate methods for statistical combination of results? RCTs; NRSI 12. If meta-analysis was performed, did the review authors assess the potential impact of RoB in individual studies on the results of the meta-analysis or other evidence synthesis? 13. Did the review authors account for RoB in individual studies when interpreting/discussing the results of the review? 14. Did the review authors provide a satisfactory explanation for, and discussion of, any heterogeneity observed in the results of the review? 15. If they performed quantitative synthesis did the review authors carry out an adequate investigation of publication bias (small study bias) and discuss its likely impact on the results of the review? 16. Did the review authors report any potential sources of conflict of interest, including any funding they received for conducting the review? ABBREVIATIONS: CI, confidence interval; MD, mean difference; RR, risk ratio; SMD, standardized mean difference. AMSTRAR domain responses: N—not answered; N-MA no meta-analysis conducted; PY—partially answered; PY RCT (include only RCT); Y—yes fully answered; Y Both—(RCT and NSRI); Y RCT (include only RCTs); AMSTAR Grading: High certainty (H): we are very confident that the true effect lies close to that of the estimate of the effect; Moderate certainty (M): we are moderately confident in the effect estimate: the true effect is likely to be close to the estimate of the effect, but there is a possibility that it is substantially different; Low certainty (L): our confidence in the effect estimate is limited: the true effect may be substantially different from the estimate of the effect; Very low certainty (VL): we have very little confidence in the effect estimate: the true effect is likely to be substantially different from the estimate of effect.

## Data Availability

We abide by the data sharing policy.

## Appendix A

**Table A1:** Summary (characteristics) of intervention types and component integration of studies included in the umbrella narrative review and from historical gap studies.

Author (Year) Country	Study			Intervention Content (IC)	Intervention Measures	
	Type, n	Participants Database		1. Aims/2. Design/3. Population/4. Care Domains and Intensity	@ Delivery Domain 1. DF; 2. DP	Outcomes 1. Primary; 2. Secondary
Zhao et al., 2024 China [18]	SR RCT n = 19	P-4681 R: 10–1518 FU: 3–12 m	Em, Md, Pb, SD, WS In to 2022	1. Evaluate HF models of care delivering multiple DMP interventions. 2. Outpatients and ambulatory 3. IR: patient; IC: SM, HF education, rehab exercise, depression	1. Tele, IT based, home 2. MDT; Nurse-led; education program; Allied health structured	1. MACE 2. SM knowledge and behaviors; HRQoL, anxiety, depression; healthcare resourcing
Chen et al., 2023 Taiwan [19]	SR+MA RCT N = 13	P = 2666 R: 28–197 FU: 2–24 m	CI, CL, Pb, Md 2002–22	1. Effects of collaborative health management team on HF 2. Hospital, Outpatients and ambulatory 3. IR: patients IC: Components—Patient-Centered Disease Management (PCDM), Collaborative Care (CASA), Integrated primary/secondary care, Multidisciplinary, DMP outpatient clinic, HF program, nurse-led, Collaborative care model (CCM), Collaborative MDT nursing team	1. F2F, Gp. media, apps, written 2. Collaborative health team: Nurse, cardiologist, psychiatrist, primary care physician, palliative care physician, social worker	1. MACE 2. Mood, HFQ0L, 6MWT; SM knowledge and behaviors; Healthcare resourcing
N’Li 2023 China [20]	SR+MA RCT N = 10	P = NA R = NA FU 3–12 m	CE, CK, Eb, Pb, WoS In to 2022	1. Transitional care strategies and improved HRQoL 2. Hospital, outpatient, ambulatory 3. IR: patients; IC: nurse-coordinated DMP, home telemonitoring, HITH, education, traditional Chinese medicine	1. Tele, Internet based, home visits 2. Nurse	1. SM behavior and knowledge 2. HRQoL, MACE, Healthcare resourcing
okYang 2023 Mly [21]	SR+MA RCT N = 105	P = 37,607 R = NA FU 6–12 m	CL, Em, Ov, Pb In - 2022	1. Effectiveness of multicomponent integrated care on clinical outcomes 2. Hospital, outpatients, ambulatory 3. IR: patient, carer; IC: case management, team change, E-health, facilitated relay, continuous QI, audit and feedback, clinician education and reminder, patient–provider communication, patient education and reminder system, promotion of SM	1. Variable see IC 2. Variable see IC	1. MACE 2. Healthcare resourcing
Hafkamp 2022 Holland [22]	USR SR+MA RCT N = 44/186	P = 6101 R = 40–1650 FU: 3–34 m	CI, CL, Pb, PsI WoS 2011–2021	1. Umbrella review of SR+MA on effectiveness of interventions in reducing HF-related (re)hospitalization 2. Hospital, outpatient, ambulatory 3. IR: patients; IC: Pharmaceutical, device, rehabilitation, multidisciplinary—variable hemodynamic, m-health, nurse-led, Hemodynamic/TM, m-health, nurse-led titration, STS, CR	1. Variable see IC 2. Variable see IC	1. HFH
Hsu 2022 Taiwan [23]	SR+MA RCT/Co N = 6/1	N = 2346 R = 40–1937 FU: NA	CI, CL, CP, Em, Pb In - 2020	1. Explore the health outcomes and participation in CR of patient navigators for HF during the transition care period 2. Hospital, outpatients, ambulatory 3. IR: patient; IC: Patient navigator program (PN) (persons with or without a healthcare background, who assists patients in CDSM e.g., nurse, peer, personal, health navigators, etc.); Transitional care interventions of health literacy, SM skills, continuity of care.	1. Written, Tele, home I 2. Nurse, navigator	1. Rehospitalization, 2. Healthcare resourcing
Toback 2017 Canada [24]	SR RCT+NR N = 26	NA	Pb, UP 1999–2016	1. Investigate educational, behavioral and psychosocial strategies that plays an important role to improve patient SM. 2. All domains 3. IR: patients: IC: variable SM	1. Variable 2. Variable	1. Compliance, 2. HRQoL, MACE, healthcare resourcing
Taylor 2005 UK [25]	SR RCT N = 21 (16 RCT)	P = 1627 R: 34–1200 FU: 3–12	Am, D, CI, CL, Em, Med; NHS, NRR, SCI; Si In - 2003	1. Effectiveness of DMP interventions for patients with HF. 2. Hospital, outpatient, ambulatory 3. IR: patients; IC: Clinical service intervention: Multidisciplinary model; Case management models; Clinic models. Educational programs	1. Written, F2F, Tele 2. Variable	1. MACE 2. HRQoL, healthcare resourcing
Roccaforte 2005 Canada [26]	SR+MA RCT N = 33	P = 7538 R = 34–1518 FU: 3–22 m	CL; Em; Med; Pb 1980–2004	1. Reevaluate the effectiveness of HF DMP on MACE and outcomes 2. Hospital, outpatient, ambulatory 3. IR: Patient and family education; IC: education improvement in patient adherence to drug therapy education, discharge plan, counselling.	1. F2F, home, tele, monitor 2. MDT (cardiologist, physician), case management (nurse, pharmacist, case manger)	1. MACE
Gonseth 2004 Spain [27]/[88]	SR+MA RCT [21]/Co [21] N = 54	P=>19030 R: 34–1966 FU: 1–50.4 m	CL, Em, Med 1966–2003	1. Evaluate DMPs reducing hospital re-admissions among elderly Effectiveness of disease management programs 2. Hospital, outpatient, ambulatory 3. IR: patients; IC: DMP: SM Education, counselling, and monitoring, diet, and drug therapy compliance. Early discharge planning and coordination of care, timely medical visits, surveillance. Structured patient education, intervention	1. F2F, tele 2. MDT, Nurse, Phar, CM	1. MACE 2. HRQoL
Huang 2023 Taiwan [28]	SR+MA RCT N = 25	P = 2746 R 40 to 228 FU: 1 w–12 m	CE, CI, Em, Med, PsI, WoS In to 2022	1. Effects of nurse-led self-care interventions 2. Hospital, Outpatients, ambulatory IR: patients; IC: HF, SC education,	1. F2F, Tele 2. Nurse	1. MACE; HRQoL; Health resourcing
Nwosu 2023 UK [29]	SR N = 18	P = 2413 FU: 3–24 m	CI, Med, PsI, WoS In to 2022	1. Nurse-led patient education programs on the quality of life 2. NA 3. IR: Patient; IC: in-person nurse-led patient education	1. Mixed F2F, audio, digital, telephone, email 2. Nurse	1. HRQoL Mortality
Checa 2022 Spain [30]	SR+MA RCT+QE, Co N = 30	P = 8209 R-24 to 1894 FU: 1–12 m	CE, CL, CT, Em, ICTRP, Med, WHO In to 2022	1. Effect of nurse-led case management models on an advanced HF 2. Hospital, Outpatients, ambulatory 3. IR: patients; IC: Telemedicine and home-visit interventions	1. Tele, TeleM, home-visit 3. Nurse	1. HRQoL 2. MACE, SM behavior and knowledge, Health resourcing
Huang 2022 Taiwan [31]	SR+MA RCT N = 24	P = 2488 R-36 to 382 30 d–12 m	CE, CI, Em, Med, PsI, WoS In to 2021	1. Effectiveness of nurse SM interventions in CHF; identify the optimal characteristics of effective nurse-led HF programs 2. Hospital, Outpatients, ambulatory 3. IR: patient; IC: nurse-led SM interventions (HF educational, skills training, psychosocial strategies, motivational interviewing, goal-setting, problem-solving, action-planning, coping strategies, emotional support, etc.)	1. Home, individual, group; F2F, tele; mail, digital, TeleM, media, written 2. Nurse-led	1. SM behaviors, knowledge
Ceu 2022 Portugal [32]	SR RCT [3], NRT N = 9	NA	CI, Med	1. Which nursing interventions help to improve basic human needs (BHN), relief of symptoms, and aid the transition from hospital to home? 2. Hospital, Outpatients, ambulatory 3. IR: patients; IC: nursing interventions. SM, health education	1. F2F, Home, Tele 2. Variable	1. HRQoL
Imanuel Tonapa 2022 Taiwan [33]	SR+MA RCT N = 12	P = 1938 R: 36–1437 2 m–12 m	CI, CL, Em, Med, Ov, Pb, WoS In to 2020	1. Effects of nurse-led telecoaching on CHF >50% of interaction 1–14 sessions 2. Hospital, outpatients or ambulatroy 3. IR: patient; IC: Motivational interview, CBT, educational, counselling	1. F2F, tele, monitor 2. Nurse?	1. HFRQoL 2. SM behavior and knowledge
Son 2020 Korea [34]	SR+MA RCT N = 8	P = 1979 R: 88–412 3 m–12 m	CI, CL, Em, Pb, WoS 2000 to 2019	1. Evidence on the effectiveness of nurse-led CHF self-care education on health outcomes amd improved self-care in CHF 2. Hospital, Outpatients, ambulatory 3. IR: patients IC: HF disease management and self-management	1. Booklets, media, F2F tele 2. Nurse	1. MACE 2. HRQoL
Walsh 2017 Abs Conf Ireland [35]	SR RCT+NR N = 68	NA	CI, Pb, Med, S 2006 to 2016	1. The role of the nurse-led clinic in patient education on the outcomes of SM and QoL for HF patients 2. Hospital 3. IR: patient, caregiver; IC: education and SM intervention e.g SM skills, health assessment; knowledge, symptom recognition, SM skills, social/caregiver support and a therapeutic nurse/patient/relationship.	1. Group or individualized programs—but not specified. 2. Nurse	1. SC behavior and knowledge 2. HRQoL
Alnomasy 2023 USA [36]	SR+MA RCT N = 14	P = 2035 R: 40–767 30 d–12 m	CL; NAHL; Pb; WoS (NA)	1. A: Effectiveness of non-pharmacological interventions (NPIs) on reducing rehospitalization among patients with HF 2. Hospital, outpatients, ambulatory 3. IR: Patient; IC: pre and post discharge education-based SC	1. home visits, tele; phone calls, digital technologies, media 2. nurse-led; mostly NA	1. Rehospitalizations
Mhanna 2023 USA [37]	SR+MA RCT N = 6	P = 489 R: 41–158 30 d–12 m	CL; Em; Med; Pb In to 2022	1. Efficacy of adjunctive CBT compared to the standard of care (SOC) in HF patients with Major Depression 2. Hospital, outpatients, ambulatory 3. IR: patients; IC: CBT including SC intervention	1. F2F, tele 2. nurse; other not stated	1. Depression and anxiety 2. HRQoL, SM behaviors and knowledge; 6-MWT distance.
Olano-Lizarraga 2023 Spain [38]	SR RCT N = 8	P = 1623 R: 64–468 30 d–12 m	CI; CL; Pb; PsI; SC; 2010 to 2022	1. Intervention studies aimed at improving the person’s social skills, reducing social isolation, loneliness, increase relationships or social networks, integration, supports and participation in social activities 2. Hospital, outpatients, ambulatory 3. IR: patient, family member; peer support; IC: CBT	1. F2F, tutorial, media, written, tele, smart phone app 3. Nurse, researchers, physicians	1. Social indices 2. MACE, SC, HRQoL
Nso 2023 Jamaica [39]	SR+MA RCT N = 9	P = 1070 3–6 m	Pb; Sco, Wos	1. Efficacy of CBT for patients with HF 2. Outpatient 3. IR: patient; IC: CBT for depression	1. F2F 2. Variable	1. Depression 2. HRQoL
Balata 2023 Germany [40]	SR+MA RCT N = 7	P = 611 R: 26–158 FU: 4–32 wk	CL, Pb, Sc, WoS (In to 2022)	1. Impact of cognitive behavioral therapy (CBT) on SM and the symptoms of depression and anxiety in HF patients. 2. Hospital, outpatients, ambulatory 3. IR: patients; IC: CBT	1. F2F; tele 3. N/A	1. Depression and anxiety 3. HRQol, SM knowledge and behavior
Koikai 2023 Kenya [41]	SR RCT+NC N = 30	P = 7685 R: 50–1223 FU: NA	CL, Em, GS Pb, SD 2012 to 2022	1. Effectiveness of SM strategies in HF 2. Hospital, outpatient, ambulatory 3. IR: patient + carers IC: SM education, structured and non-structured	1. F2F, materials 2. Nurse, allied health	1. MACE, costs 2. SM knowledge and behaviors
Feng 2023 China [42]	SR+MA RCT N = 20	P = 3459 R: 39–317 FU: 3–12 m	CK, Pb, WoS, VIP 1999 to 2022	1. Influence of SM intervention on readmission rate, mortality rate, SM ability and quality of life in HF 2. Outpatient and ambulatory 3. IR: patients; IC: SM, HF education intervention HF education.	1. F2F, Media, tele, home, teleM, rehab 2. N/A	1. MACE; SC knowledge and behaviors
Nahlen Bose 2023 Sweden [43]	Meta-review (n = 7 SR) RCT = 67	P = 10,132 R = 320 to 3837 FU: NA	CI, CL Pb, PsI In 2022	1. Meta-review on efficacy from synthesized SR-MA of psychosocial interventions in HF on outcomes 2. NA 3. IR: patients; IC: Psychoeducative component, e.g., cognitive behavioral therapy (CBT) or coping skills training (stress management);	1. NA 2. NA	1. MACE 2. HRQoL, Depression, SC, 6MWT
Lee/Reigel 2022 USA [44]	MA RCT N = 27	P = 6950 R = NA FU = NA	CI, Em, Pb, PsI 2008–2019	1. Effectiveness of SC interventions on relevant outcomes 2. Outpatient and ambulatory 3. IR: patients with six chronic conditions, carers; IC: see@	1. F2F individual/group, web, media, print, phone, tools 2. multiple see@	SC knowledge and behavior, HRQOL
Villero-Jimenez 2022 Spain [45]	SR RCT+NC N = 12	P = 1380 R = 19–369 FU = NA	CI, Pb, PsI (NA)	1. Identify dyadic SM interventions in hospital settings 2. Hospital 3. IR: patient carers; IC: cognitive, affective and behavioral	1. Variable delivery format dimensions and strategies 2. Nurse	1. Depression, Anxiety, SC, adherence 1. MACE, HRQoL
Ghizzardi 2022 Italy [46]	SR+MA RCT N = 9	P = 1214 R-30 to 510 FU = 1–16 m	CI, Em, Pb PsI, Sc In to 2020	1. Motivational interviewing (MI) in enhancing in HF patients SM 2. Hospital, outpatients, ambulatory 3. IR: patients; IC: Nurse education on SC behaviors, disease management and responses to symptoms	1. F2F, Tele 2. Nurses, researchers	1. SC knowledge and behaviors 2. HRQoL
Suksatan 2022 Thailand [47]	SR RCT+NC N = 15	P = 10,701 R-36–2494 FU: 30 d	CI, CL, Pb, PsI, SC 2011 to 2022	1. Effect of transition care interventions (TCI) on rehospitalization before discharge from hospital to home 2. Hospital, outpatients, ambulatory 3. IR: HF patients; IC: Discharge coordination, SC and HF education	1. F2F, home visit, tele, mob app 2. nurses, pharmacists, MDT	2. Rehospitalization
Meng 2021 China [48]	SR+MA RCT N = 8	P = 1707 R: 20–902 FU:6–42 m	CL, CNKI, Em, Pb 2000 to 2020	1. Efficacy of SM as an effective method to progress self-care ability 2. NA. 3. IR: patients; IC: Self-management support programs	1. F2F, Web, Tele; smartphone; per SM support program 2. NA	1. KAP model assessment of SM knowledge and behaviors
Tinoco 2021 Brazil [49]	SR+MA RCT+NR N = 19	N = 1841 R:10–475 FU: NA	CI, LI, Pb, Sc (2012 to 2019)	1. Effectiveness of health education interventions in SC and adherence to treatment of patients with HF 2. Hospital, outpatients, ambulatory 3. IR: patients, family IC: self-management education; CBT; cultural adaptations, lifestyle coaching, motivational interviewing	1. Tele, Booklet, Home, Group, Web, Media, TM 2. Nurse, Physicians, Health educator; MDT	1. SC behavior and knowledge
Aghajanloo 2021 Iran [50]	SR+MA RCT+NR N = 39	P = 8958 R: 17–2082 FU:NA	Em, GS, Ma, Pb, SID, WoS, 2004 to 2018	1. Effectiveness of SC in patients with HF through the SCHFI scale. 2. Hospital, outpatients, ambulatory 3. IR; patients; IC: SC education	1. F2F 2. Research staff	1. SC behavior and knowledge
Cañon-Montañez 2021 Colombia [51]	SR+MA RCT N = 45	P = 9688 R: 37–1049 FU: 3–18 m	CI, CL, Em, Li, Pb, Sc, WoS (In to 2019)	1. Estimate the combined effect of educational interventions (EI) on decreased readmissions and time of hospital stay in adults with HF, 2. I, outpatient, ambulatory 3. IR: patient IC: SC education	1. Visit, Tele, TM, Media, booklet 2. Nurse, other	1. HFH 1. Readmission
Anderson 2021 UK [52]	SR RCT+QE N = 12	P = 3887 R: 25–1023 FU: 3–24 m	BNI, CI, Em, Med (2008 to 2020)	1. Impact of specialist advanced nurse-led care on clinical outcomes, quality of life and satisfaction compared to physician-led care. 2. Hospital, outpatients, ambulatory 3. IR: patients; IC: HF, SC education and DMP	1. F2F, tele, home 2. Advanced-level and specialized nurses, physicians	1. MACE 2. HRQOL Quality of life; SC knowledge and behavior
Zhao 2021 China [53]	SR+MA RCT N = 15	P = 2630 R: 28–475 FU: NA	CL, Em, Pb, WoS (In to 2019)	1. Effects of self-management interventions on HF knowledge, quality of life, and HFH 2. Hospital, outpatients, ambulatory 3. IR: patients; IC: SM education, CBT, disease management	1. DF: F2F, tele, Web, booklet, home visit 2. Nurse, ONS	1. HF knowledge, 2. HRQoL, HFH
Poudel 2020 USA [54]	SR RCT+NR N = 8	P = 758 R: 30–241 FU: NA	CI, CL, GS, HS, Med, PsI (1990 to 2019)	1. Conduct an exploration and report of evidence and gaps in the literature regarding the impact of MI on HF outcomes. 2. Outpatient, ambulatory 3. IR: patient; IC: motivational interviewing	1. F2F, Tele; written materials, media, web, telemonitoring 2. Any healthcare provider type.	1. MACE 2. HRQoL
Świątoniowska-Lonc 2020 Poland [55]	SR+MA RCT N = 16	P = 944 60–1160 FU: 1–18 m	Med, Pb, Sc (2010 to 2019)	1. Role of health education in HF treatment and its impact on outcomes in patients with chronic HF 2. Ambulatory 3. IR: Patients; IC: SC Education delivered by multiple formats.	1. Tele, Home, F2F, written material, lectures, monitoring 2. Nurse, GP, specialist, dietician, pharmacist	2. HRQoL, compliance, SC behavior, rehospitalization
Peng 2019 China [56]	SR+MA RCT N = 8	P = 480 R: 17–158 FU: NA	CL, Em, Pb (In to 2018)	1. Efficacy of cognitive behavioral therapy for alleviating depression for CHF 2. Inpatient, outpatients, ambulatory 3. IR: Patients; IC: CBT	1. F2F, Web, Tele 2. Nurse, Researcher	1. Depression Scale 1. HRQoL; self-care knowledge and behavior, 6MWT
Parajuli 2019 Australia [57]	SR+MA RCT N = 18	P = 4630 R:34 to 2169 FU: 3–55 m	CI, CL, Em, Med, Pb, SC, WoS (In to 2017)	1. Evidence for the role of the pharmacist within the MDT for HF management to improve clinical outcomes 2. Inpatient, outpatients, ambulatory 3. EG: IR: patient; IC: pharmacist(s) working in collaboration, with a physician, other health-care professionals on collaborative medication management and education, discharge counselling.	1. Tele, home medication review, self-adjustment of diuretic 2. Pharmacist, physician (± MDT)	1. HFH, HFM 2. MACE, HF and SC behavior and knowledge, costs
Shanbhag 2018 Canada [58]	SR RCT+NR N = 38	P = 76,582 R = 68–50,678 FU: NA	CI, CL, Em, Med 1990–2017	1. Implementation interventions that improve physician adherence to these recommendations 2. Inpatient, outpatients, ambulatory 3. IR: Patient: IC: GDMT, Process of care, SC education	1. F2F, Web, EM-ep 2. Physician, Nurse	1. Proportion of eligible patients offered GDMT SC education; MACE
Sterling 2018 USA [59]	SR RCT+NR N = 6	P = 75,320 R-40–74,580 FU: 1–12	AgeLine, CI, CL, Em, Med In-2017	1. HCW on HF management 2. Inpatient, outpatients, ambulatory 3. IR: Patient; IC: educational intervention	1. Variable 2. HW	1. HFH 2. Health economics
Jiang 2018 Taiwan [60]	SR+MA RCT N = 29	P = 3837 R: 23–902 FU: NA	CI, CL, Em, Pb, PsI, SC, WoS, ProQ 2006–2016	1. Effectiveness of RCT using psychological methods on SC behaviors, 2. Outpatients 3. IR: patient; IC: CBT	1. F2F and gp, tele, 2. Doctors, nurses, allied health, researchers.	1. Self-care behaviors, 2. Anxiety, depression, HRQoL, physical function., healthcare resourcing
Jonkman 2016 Holland [61]	MA RCT N = 20	P = 5624 R: 42–1023 FU: 3–18	CI, CL, Em, Pb, PsI 1985–2013	1. Characteristics of self-management interventions effective in influencing HRQol, mortality, and hospitalizations 2. Inpatient, outpatients, ambulatory 3. IR: Patient, Family/Carer/Partner; IC: SMI	1. F2F, Tele, TM, EMep, HV 2. N, Phy, Pha, HW	1. ACM, 2. ACH, HFH, HRQoL
Ruppar 2016 USA [62]	SR+MA RCT+NC N = 57	P = 4527 R:10–1518 FU: NA	CI, CL, D, IPA, Highw, Med, Sc, PQ In-2013	1. Interventions to improve medication adherence 2. Inpatient, outpatients, ambulatory 3. IR: Patient, carers; IC: medication education and disease education;	1. F2F, Tele, TM, txt, web, Media, PM 2. MD, nurses, phar, phy, diet, SW, CM, HCW	1. ACM 2. ACH, HFH
Jonkman 2016 Holland [63]	SR+MA RCT N = 20	P = 5624 R: 42–1023 FU: 3–18	CI, CL, Em, Pb, PsI 1985–2013	1. Self-management interventions efficacy in influencing MACE 2. Inpatient, outpatients, ambulatory 3. IR: Patient, Family/Carer/Partner; IC: SMI	1. F2F, Tele, TM, EMep, HV 2. N, Phy, Pha, HW	1. ACM, 2. ACH, HFH, HRQoL
Srisuk 2016 Thailand [64]	SR RCT N = 9	P = 666 R: 61–155 FU: 5–24 wk	CI, CL, Em, Med, Pb, PsI, Sc, WoS 2005–2015	1. Interventions to improve medication adherence. 2. Inpatient, outpatients, ambulatory 3. IR: Patient, carers; IC: medication education and disease education;	1. F2F, Tele, TM, txt, web, Media, PM 2. Nurse, Res, MD	1. SC knowledge. behavior 2. HRQoL; readmission
Ha Dinh 2016 Vietnam [65]	SR RCT+NR N = 12	P = 467 R: 88–276 FU: 12–15	CI, CL, Em Med, WoS In - 2013	1. Teach-back method in health education programs for improving adherence and self-management 2. Inpatient, outpatients 3. IR: patient; IC: Teach back method	1. F2F 2. RN; Phar, HCW	1. SC knowledge, behavior 2. HRQoL, readmission
Inglis 2015 Australia [66]	SR+MA RCT N = 41	P = 9332 R: var FU: var	Am, CE, DARE, HTA, Med, Em, CI, SCI, In - 2014	1. Heart failure management delivered via structured telephone support 2. Inpatient, outpatients, ambulatory 3. IR: patient IC: disease management	DF: All options 2. DP: All options	2. MACE
Ruppar 2015 USA [67]	SR RCT+NR N = 29	P = 4285 R: 10–902 FU: 1–24	CI, CL, Em, Med In - 2013	1. Quantify the effect of interventions to improve adherence to HF medications 2. Inpatient, outpatients, ambulatory 3. IR: patient; IC: Medication and disease education	1. Verbal (F2F, Tele, TeleC), written/electronic, video Nurse, pharmacists	1. SC Behavior
McGreal 2014 USA [80]	SR RCT N = 9	P = 1415 R: 44–605 FU: 3–12 m	CI, Med, Pb, CINAHL, 2010–2014	1. Examine the effectiveness of HF self-care interventions in relation to clinical events and symptom burden. 2. Inpatient, Outpatients, Ambulatory 3. IR: patient; IC: variable HF, SC education	1. F2F, home; monitoring, Media, tele 2. Nurse, allied health	1. SC Behaviors. Knowledge MACE
Casimir 2014 [68]	SR RCT NT = 7	P =1260 R: 121–314 FU: 1–12 m	CEN, CI, CL, EM, ERIC, JBI, Med Inc to 2010	1. Heart failure on knowledge, self-care behaviors, quality of life, and readmissions. 2. Inpatient, outpatients, ambulatory 3. IR: patient; IC: SC and HF education	1. F2F, written 2. Nurse; MDT	1. HF knowledge, 2. SC behaviors; HRQoL; Readmissions
Wakefield 2013 USA [69]	SR+MA RCT N = 43	P = 8071 R: 25–1518 FU: 3–18 m	CI, CL, Med 1995–2008	1. Quantify individual interventions used in multicomponent outpatient HF management program 2. Outpatient 3. IR: Patient, caregiver; IC: Variable education and intervention	1. F2F, TeleM, TeleH, Tele, msg 2. Allied health	1. MACE 2. SC knowledge and behavior; HRQoL
Barnason 2012 USA [70]	IR RCT N = 19	P = 3166 R: 18–902 FU: 2–36 m	CI, CL, Med, PsI	1. Examine the interventions used to improve self-care of HF patients 2. Inpatient and outpatient 3. IR: patient; IC: CBT	1. F2F, Gp, Media, Tele, TeleH, 2. Nurse	1. MACE 2. SC knowledge and behaviors; HRQoL
Boyde 2011 USA [71]	SR RCT N = 19	P = 2686 R: 36–314 FU: 3–18 m	CI, CL, Em, Med, PsI	1. Educational interventions that have been implemented for HF patients and to assess their effectiveness 2. Outpatient 3. IC: education; IR: HF patients	1. F2F, pEM, Media 2. Nurse	1. HFH 2. SC knowledge, behaviors, HRQoL
Dickson 2011 USA [72]	MA RCT N = 3	P = 99 R: NA FU: NA	Med, Pb	1. Explore how comorbidity influences HF self-care. 2. Outpatient 3. IC: questionnaire; IR: HF patients	1. pEM 2. Allied health	2. SC behaviors
Yehle 2010 USA [73]	SR RCT+NR N = 12	P = 1747 R: 20–801 FU: 1–12 m	CI, CL, ERIC, Med, Pb	1. How to structure educational interventions for heart failure patients to improve their self-efficacy for self-care behaviors 2. Outpatients 3. IR: patient; IC: HF and SC education	1. Tele; monitoring device, pEM 2. Allied health	1. SC Knowledge
Ditewig 2010 Holland [74]	SR RCT N = 19	P = 4011 R: 50–766 FU: 6–24 m	CI, CL, Em, Med	1. Effectiveness of self-management interventions compared to usual care 2. Hops, Op, Ab; EF = HFrEF 3. IR: Patient; IC: Structured HF, SC Education	1. Tele, Media, Video, pEM 2. Nurse, Phar, CM, MDT	1. MACE 1. SC Behaviors; HRQoL
Boren 2009 USA [75]	SR RCT N = 35	P = 7413 R: NA FU: NA	CI, CL, Med	1. Identify educational content and techniques that lead to successful patient self-management and improved outcome 2. Outpatients 3. IR: patient; IC: Structured education knowledge, social interaction and support, fluid management, diet and activity	1. Multiple 2. Allied Health	1. SC Behaviors 2. HRQoL
Jovicic 2006 Canada [76]	SR RCT N = 6	P = 857 R: 70–223 FU: 3–12 m	ACP, CI, CL, Em, Med	1. Effectiveness of self-management interventions 2. Hosp, Outpatient 3. IR: Patient, family IC: Structured Education	1. F2F, Tele, SMS, Media 2. Nurse, Phy	1. MACE 2. HRQoL
McAlister 2004 Canada [77]	SR RCT N = 29	P = 5039 R: 34–1396 FU: 1–12	AMED, CI, CL, Em, Med	1. Multidisciplinary strategies improve outcomes for heart failure 2. Hosp, Outpatient 3. IR: Patient: IC: Structured patient education	1. Tele, TM, Media 2. MDT, Nurse, Phar, CM	1. MACE 2. HRQoL; Cost

**Table Glossary and Abbreviation:**

Databases Abbreviations: Am = AMED; BNI = British Nursing Index; CE = (CENTRAL) Cochrane Central Register of Controlled Trials; CI = (CINAHL) Cumulative Index to Nursing and Allied Health Literature; CK = CNKI; CL = Cochrane Library; CP = Chinese Electronic Periodical Services; D = DARE; DMP = disease management programs; Dy = DynaMed; Eb = EBSCO; Em = (Embase) Excerpta Medica Database; ERIC = Education Resources Information Center; GS—Google Scholar; HF—heart failure; HS—Health Source/Nursing/Academic Edition; ICTRP—Registry of International Clinical Trials; IPA—International Pharmaceutical Abstracts; LI—LILACS; Ma—Magiran; Med = Medline; National Research Register (July 2003); NAHL—Nursing and allied Health Literature; NHS Economic Evaluations Database; OP—hospital outpatients; Ov = Ovid; Pb = PubMed; pEM—printed educational material; PQ—ProQuest; PsI = PsycInfo; S = Science Direct; SC = Scopus; SCI = Science Citation Index; Si = SIGLE; SID = Scientific Information Database; Up-To-Date; WHO—World Health Organization; VIP = Wan Fang, Wei Pu; WoS = Web of Science.

Table abbreviations: EF—ejection fraction; Exp Gp—experimental group (IR—intervention received; IC—intervention content); MACE—major adverse cardiovascular events; MDT—multidisciplinary team; n—number of studies; QOL—quality of life; RCT—randomized controlled trial; SM—self-management.

Health resourcing Abbreviations: Health services (QALY), cost, effectiveness, utilization of individual health journey domains; health economics; DMP: counselling, and monitoring to enhance self-control mechanisms, timely medical visits, diet, and drug therapy compliance. Early discharge planning and coordination of care, individualized and comprehensive patient and family HF education, telephone follow-up and surveillance, promotion of optimal HF medications and medication doses based on consensus guidelines; SM:—self-care components, education, skills knowledge, behaviors, monitoring, theories; SCHFI on Goal setting and motivational interviewing, educational and self-care needs, barriers and facilitators; MACE—one component of readmission, mortality, either general or heart failure related; Physical function—6 MWT; Intervention: Facilitated, Education, Self-management, Weight-monitoring, Sodium diet advice/restriction, Exercise recommendation, Medication review, Social and psychological support education, discharge plan, counselling.

*Intervention Category Description:*

- Case Management—Based on implementation of a collaborative process between one or more care coordinators or case managers and the patient, to assess, plan, and facilitate service delivery for patients with chronic diseases, particularly when transitions across healthcare settings are required.
  ○ The Collaborative Health Management—is a model that builds on teamwork between the nurse practitioners and physicians, as an egalitarian partnership. Its long-term purpose is to operationalize Advanced Practice Registered Nurses to deliver high-quality chronic disease management.
  ○ Transitional Care—The support provided to patients as they from one phase of disease or its management, such as from hospital-based care to home based care. This can involve both patients and families and with a range of medical emotional and other needs as they adjust and attain their care goals.
  ○ Multicomponent Integrated Care—Coordination between different healthcare providers, providing patient education and self-management support, and facilitating smooth transitions between hospital and home care settings.
  ○ Care Pathways—e.g., Telemonitoring, Structured telephone support, MDT clinic, cardiac care clinics (center based: individual or disease management programs; rehabilitation; community-based follow-up).
- Chronic Care Model—Model that identifies six modifiable elements of healthcare systems: (1) organizational support, addressing organizational culture and leadership, (2) clinical information systems to organize patient, population and provider data, (3) delivery system design to address composition and function of the care team and follow-up management, (4) decision support to increase provider access to evidence-based guidelines and specialists for collaboration, (5) self-management support to provide tailored education, skills training, psychosocial support and goal-setting, and (6) community resources to provide peer support, care coordination, and community-based interventions.
- Discharge Management—Interventions designed to facilitate effective transitions from hospital care to other settings. Typically includes a predischarge phase of support, transitional care for the move between the hospital and community/home setting, and post discharge follow-up and monitoring, often incorporating rehabilitation or reablement support.
- Complex Interventions—Two reviews assessed a range of interventions rather than focusing on a single intervention or service model.
- Multidisciplinary Team—Interventions comprising teams composed of multiple health and/or social care professionals working together to provide care for people with complex needs. Teams typically included condition-specific expertise, nurses, occupational therapists, physiotherapists, social workers, GPs, and occasionally pharmacists or case managers.
- Disease Management—Six components: 1. Population identification process, 2. Evidence-based practice guideline, 3. Collaborative practice models, 4. Patient self-management education, 5. Process and outcome management, 6. Reporting and feedback loop.
